# Translation and validation of the Functional Assessment of Cancer Therapy-Bone Marrow Transplant (FACT-BMT) version 4 quality of life instrument into Arabic language

**DOI:** 10.1186/s12955-018-0861-7

**Published:** 2018-03-12

**Authors:** Hussein Soudy, Irfan Maghfoor, Tusneem Ahmed M. Elhassan, Eman Abdullah, Shahzad M. Rauf, Ahmed Al Zahrani, Saad Akhtar

**Affiliations:** 1 Oncology Center, King Faisal Specialist Hospital & Research Center, P.O. Box 3354, MBC# 64, Riyadh, 11211 Kingdom of Saudi Arabia; 2 Department of Nursing Affairs, King Faisal Specialist Hospital & Research Center, Riyadh, Kingdom of Saudi Arabia

**Keywords:** Fact-BMT, Quality of life, Arabic translation, Translation and validation, Bone marrow transplant, Stem cell transplantation, Autologous stem cell transplantation, Lymphoma, Hodgkin lymphoma, Non-Hodgkin lymphoma

## Abstract

**Background:**

Functional Assessment of Cancer Therapy-Bone Marrow Transplant (FACT-BMT) has been translated from English into several languages. Currently, there is no validated translation of FACT-BMT in Arabic. Here, we are reporting the first Arabic translation and validation of the FACT-BMT.

**Methods:**

The study was approved by the Institutional Research Advisory Council. The Arabic translation followed the standard Functional Assessment of Chronic Illness Therapy (FACIT.org) translation methodology (with permission). Arabic FACT-BMT (50- items) was statistically validated. Cronbach’s alpha for internal consistency, Spearman’s rank correlation coefficients method for Inter-scale correlations and Principal Component Analysis for factorial construct validity was used.

**Results:**

One hundred and eight consecutive relapsed /refractory lymphoma patients who underwent high dose chemotherapy and autologous stem cell transplant were enrolled. There were 68 males (63%) and 40 females (37%) with a median age of 29 years (range 14–62). After Arabic questionnaire pre-testing (Cronbach’s alpha 0.744), the study included 108 patients. Cronbach’s alpha for the entire FACT-BMT indicated an excellent internal consistency (0.90); range (0.67 to 0.91). Cronbach’s alpha for sub-groups of social (0.78), emotional (0.67) and functional wellbeing was (0.88). Cronbach’s alpha for bone marrow transplant (0.81), FACT-General (0.89), and FACT- Trial Outcome Index (TOI); (0.91) also revealed excellent internal consistency. Patients had high scores in all domains of quality of life, indicating that most patients were leading a normal life. This translation of FACT-BMT in Arabic was reviewed and approved for submission by the FACIT.org.

**Conclusions:**

Our data reports the first translated, validated and approved Arabic version of FACT-BMT. This will help large numbers of Arabic speaking patients undergoing stem cell/bone marrow transplantation, across the globe.

**Electronic supplementary material:**

The online version of this article (10.1186/s12955-018-0861-7) contains supplementary material, which is available to authorized users.

## Background

Non-Hodgkin lymphoma (NHL) and Hodgkin lymphoma (HL) constitute 6.4% and 3.4% of all cancers in Saudi Arabia, respectively [1]. Together, lymphoid malignancies (HL and NHL) are the second most common cancer (NHL being the 4th and HL 8th) in Saudi Arabia. Patients with refractory or relapsed NHL and HL can undergo potentially curative therapy with high dose chemotherapy (HDC) and autologous hematopoietic stem cell transplantation (ASCT) [2, 3]. HDC ASCT requires stem cell mobilization as well as prolonged hospitalization for HDC and recovery of the bone marrow function. These result in significant risk of both short and long term treatment related morbidity [4]. In addition, owing to increasing numbers of HDC ASCTs and increasing numbers of survivors, long-term effects of HDC on the quality of life (QoL) of patients and family members are becoming important. QoL has been defined as “the functional effect of an illness and its consequent therapy upon a patient, as perceived by the patient” [5]. For a long time, QoL related research and outcomes faced issues related to lack of consensus on the definition of the main concepts and standardization of assessment tools [6, 7]. QoL measures include general and disease-specific questions. Although general measures of QoL can be used for various groups of patients, disease-specific QoL includes aspects of health (symptoms, impairments, and disabilities) that are relevant to a particular disease.

Functional Assessment of Cancer Therapy - Bone Marrow Transplantation (FACT-BMT) is a simple, brief and self-administered questionnaire that was originally developed and validated in the English language in the US by McQuellon et al. [8]. FACT-BMT is a combination of two tools; functional assessment of cancer therapy-general (FACT-G) [9] assesses the effects of cancer therapy in the four major areas of physical, social/family, emotional and functional well-being that has been described and validated previously in a number of studies [9–12]. The bone marrow transplant sub-scale (BMTS) assesses specific BMT related concerns and constitutes the second part of FACT-BMT. This has been developed in collaboration with patients and experts in the field with the primary aim of capturing information on problems specifically related to hematopoietic stem cell transplantation. The FACT-BMT has been translated and validated in several languages including Spanish, Dutch, French, German, Italian, Norwegian and Swedish, Japanese, Russian, Chinese, Portuguese and Korean [13–17].

At the time of start of this study, there was no Arabic translation of FACT-BMT. The purpose of this study was to translate and validate FACT-BMT (Version 4) in Arabic and report QoL as this might be useful to assess and understand the issues related to QoL in Arabic speaking patients, not only for the physicians and researchers but also for patients undergoing HDC ASCT.

## Methods

### Study design and participants

This study was approved by the Institutional Research Advisory Council and Ethics Committee as a prospective study. Eligibility criteria included (a) age 14 years and above, (b) HDC ASCT at least a 100 days ago in the Section of Adult Medical Oncology for HL or diffuse large B cell (DLBCL), (c) no psychiatric illness and (d) able to give informed consent (in case of minors, consent obtained from legal guardian). All patients aged > 14 years are seen and managed in Adult Medical Oncology according to hospital policy. The pretest was carried out on the first 20 patients following which the cohort was extended to a total of one hundred and eight consecutive patients with lymphoma (including the pretested patients) who had undergone HDC ASCT at our institution in order to statistically validate and assess the quality of life. Almost all of these patients were uniformly treated with etoposide, solumedrol, Ara-C and cisplatin (ESHAP) with or without rituximab as salvage chemotherapy and busulphan, etoposide, Ara-C and melphalan (BEAM) [3] as conditioning regimen and were followed in outpatient lymphoma clinic at King Faisal Specialist Hospital and Research Center, Riyadh, Saudi Arabia between February 2008 through December 2013.

### Administered questionnaire

The FACT-BMT is designed for patient self-administration, either on paper or directly on the computer. It can also be administered using face-to-face or telephone interview. Interview administration is appropriate with adequate training of interviewers to minimize bias towards patient responses. In this study, we elected to use face-to-face interview owing to our lack of prior experience in translating and validating questionnaires from a different language. The FACT-BMT (Version 4) has a total of 50 questions. This is a combination of BMT domain (named as Additional Concerns) added to the FACT-G. The FACT-G is a 27-items questionnaire of general questions divided into four primary quality of life domains: physical well-being (PWB; 7-items), social/family well-being (SWB; 7-items), emotional well-being (EWB; 6-items); and functional well-being (FWB; 7-items). The BMT domain has 23 items, 18 items address the BMT related side effects, specifically designed to assess the BMT patients’ quality of life [8]. Five other questions from different QoL questionnaire were added in the BMT domain as they were considered relevant to FACT-BMT: C6 and C7 were taken from the colon cancer QoL, BL4 from the Bladder Cancer QoL, Br1 from Brain tumor QoL and B1 from Breast cancer QoL questionnaire. One lymphoma and transplant expert (S.A) also developed 17 extra cultural questions in English with the help of 3 native Arabic speaking oncologists, 1 non-Arabic speaking transplant physician and one native Arabic speaking transplant coordinator nurse. These questions represent specific social and cultural issues/concern encountered and felt important by the team in this society and also frequently asked by the patients. These 17 extra cultural questions are neither part of FACT-BMT nor related to any existing FACIT.org questioner. These questions (English and Arabic versions) are presented as supplemental data and results are presented in the results section.

### Questionnaire validation

#### Internal validity

Internal validity was utilized in order to assess the validity of the result within the study.

It measures the association between different questionnaire items and the outcome. Internal validity has been measured using Reliability coefficients Cronbach’s alpha for each separately for each of mentioned domain.

#### Construct/factorial validity

Factorial validity was applied in order to measure the correlation between a group of questionnaire items and a specific factor (construct). Factorial validity has been utilized using factor analysis. Principle component was used as an extraction method where questionnaire items have been divided into five components/factors (PWB, SWB, EWB, FWB and BMT). Equamax with Kaiser Normalization was used as a Rotation Method. Among all rotation methods, Equamax showed the best performance. However, other rotation methods showed comparable results.

### Scoring of questionnaire

Details of scoring methodology for the FACT-BMT are provided on facit.org website. FACT-BMT is scored according to its domains, as the sum of the scores for its questions. The Likert scale from zero to four [0–4] is used to measure the responses for each question after taking into account reverse scores for questions constructed in a negative form. The final score for FACT-BMT ranges from zero to 196 [18]. Higher scores for the scales and subscales indicate better quality of life. FACT-G which is a total score PWB, SWB, EWB and FWB sub-scores. FACT-BMT which is a total score of PWB, SWB, EWB, FWB and selected items in BMT sub-scores.

Trial Outcome Index (TOI) is the sum of the PWB, FWB and “additional concerns” subscales / BMT sub-scores. The TOI is an efficient summary index of physical/functional outcomes. TOI is likely to change quickly over-time or due to therapy. This endpoint is also commonly used in clinical trials because it is responsive to change in physical and functional outcomes, sometimes more than a total (overall) multidimensional aggregated score which includes SWB and EWB.

### Translation process of FACT-BMT into Arabic version

The original FACT-BMT was developed in English using a standardized approach for item derivation, reduction, and testing and has been used extensively in various clinical trials.

#### Translation Methodology

The Arabic translation followed the standard Functional Assessment of Chronic Illness Therapy (FACIT) translation methodology as described at FACIT.org. Seventeen extra questions were also translated using the same setting and methodology along with the Arabic translation of FACT-BMT (Version 4). The translation process included following steps:

Step one**:** Involved forward translation from English FACT-BMT (Version 4),marked as English version 1 and obtained from FACIT.org after permission, to Arabic by two Arabic speaking people, one oncologist (Saudi Arabian) and other oncology health educator (Egyptian) specialized in educating patient undergoing HDC ASCT; both were fluent in English and aware of the research objectives. The third translation was also performed by a commercially available certified translation service with no knowledge of the project. These translations resulted in three ‘Arabic Versions 1 (A, B, C)’.

Step two: Reconciliation of these three Arabic Versions [1] (A, B, C) was performed by bilingual hospital nursing educator. Minor corrections were made and resulted in ‘reconciled Arabic version 1’.

Step three: Backward translation of the reconciled Arabic Version 1 into English, named ‘English Version 2’ by bilingual people fluent in both Arabic and English languages and were aware of the study objectives.

Step four: Independent review of the reconciled Arabic Version 1 and English version 2 by three bilingual experts; one physician and two patient’s coordinators with medical knowledge reviewed the translation independently and made suggestions if needed. Minor grammatical changes were made according to their recommendations.

Step five: Meeting of primary investigators to discuss the findings, ensure spelling and grammatical issues. This led the generation of Arabic translation - the final version of Arabic FACT-BMT which was considered grammatically and linguistically equivalent to the original English FACT-BMT (Version 4).

Step six: Guidelines for the interview and other methods were established to ensure uniform style of administering this Arabic FACT-BMT to patients.

Step seven: FACT-BMT, Arabic version questionnaire was given to 20 native Arabic speaking patients, who were physically present to complete the study questionnaire in a pre-testing phase. All patients were fully informed about the study and how to complete the questionnaire. Each patient was then interviewed to provide comments on comprehensiveness and clarity of the items and the degree of difficulty encountered when answering the questionnaire. The pre-test results indicated excellent understanding, good content coverage, and overall comprehensibility. We continued with other patients included in the study without amendment in this Arabic FACT-BMT.

### Permission from FACIT.org

Formal permission through e-mail from a FACIT.org representative was obtained for the attached Arabic FACT-BMT. Thirty-five questions had already been translated and validated prior to our study and were already in use in “different” quality of life studies (not as a group in FACT-BMT). Nineteen out of these 35 questions displayed 95% or more match in wording and 100% match in meaning with our translation according to our bilingual native Arabic speaking team and independent reviewers. Sixteen remaining questions had a less than 95% match in wording but 100% match for the meaning according to the same team. Sixteen of twenty-three questions in “Additional Concerns” were translated and validated for the first time in our study. Questions C6, C7, BL4, BMT5, BMT6, BMT7 and B1 in the “Additional Concerns” had already been translated into Arabic from other FACIT questionnaires. After discussing with the FACIT.org representative, it was agreed that we will merge our 16 first time translated and validated questions with the previously translated and validated 35 questions for this first complete Arabic FACT-BMT questionnaire for all future use as per defined terms, conditions and guidelines of FACIT.org (available on www.FACIT.org). This Arabic FACT-BMT (Additional file 1) represents a merged version of preexisting and validated questions from prior studies and current study and was submitted to the FACIT.org representative in December 2011 as the final, Arabic translated and validated the version of FACT-BMT (Version 4). This is the version already available on www.FACIT.org. 

### Statistical methods

The Arabic-translated FACT-BMT (Version 4) was first studied by examining the internal consistency of the subscales and total scores using Cronbach’s alpha. Inter-scale correlations among the FACT-BMT domains were obtained using Spearman’s rank correlation coefficients in order to measure the correlation between individual QoL domains [19].

Separate models were developed and Cronbach’s alpha was calculated for each QoL composite (PWB, SWB, EWB and FWB) or subscale scores such as FACT-G, FACT-BMT, or TOI.

A convenient sample of 108 patients was used to evaluate the questionnaire reliability. The demographic and clinical characteristics were summarized by descriptive statistics. Wilcoxon test was used in order to compare the median scores for different FACT-BMT domains among patients < 30 years and patients ≥30 years. Statistical analyses were conducted using SAS/STAT software, Version 9.2 (SAS Institute, North Carolina, USA) [20].

## Results

Patient population: A total of 108 patients participated in this study. Details related to gender, timing of interview and educational level are shown in Patient characteristics, Table 1.

**Table 1:** Patients’ characteristics

Variable	Total Number (108)	Percentages (%)
Median age at HDC ASCT (range)	108	Median age 29 years (14–62)
Age 14–21	16	15
Age > 21–31	42	39
Age > 31–51	40	37
Age > 51–62	10	9
Time from transplant to interview^a^		
> 3–12 months	31	30.4
> 12–24 months	25	24.5
> 24–60 moths	30	29.4
> 60 months	16	15.7
Educational level^a^		
No formal education	7	6.9
Elementary school	12	11.8
Middle school	15	14.7
High school	34	33.3
Baccalaureate Degree or over	33	32.4
Sex		
Female	40	37
Male	68	63
Diagnosis		
Hodgkin lymphoma	75	69.4
Non-Hodgkin lymphoma	33	30.6

^a^Few with missing value

Pretest: Pretest was done on the first 20 patients who were first recruited in the study. The questionnaire reliability was assessed using Cronbach alpha. The test revealed a Cronbach alpha of 0.744 and the study was then extended to a total of one hundred and eight consecutive patients to evaluate the Arabic FACT-BMT questionnaire reliability. The means and standard deviations (SD) for patients with the complete set of related information were provided as measures of uncertainty for different questionnaire domains (Table 2). Additional files 1 has the complete translation as “FACT-BMT, Version 4, Arabic Translation” and Table 3 has the “FACT-BMT, Version 4, English version”. Additional files 2 and 3 are showing cultural question - Arabic and English version respectively.

**Table 2:** QoL scores for cohort members at enrollment time

FACT-BMT	Number	Median	Lower quartile	Upper quartile
Physical wellbeing (PWB)	104	24	19	27
Social wellbeing (SWB)	103	24	20	27
Emotional wellbeing (EWB)	104	20	17	22
Functional wellbeing (FWB)	103	23	19	27
Bone Marrow transplant (BMT)	104	89	76	100
FACT-General	102	66	57.5	75.7
FACT-BMT	102	154.5	134	174.3
FACT- Trial outcome Index (TOI)	103	113	95	127.5

**Table 3:** Translation and validation of the Functional Assessment of Cancer Therapy-Bone Marrow Transplant (FACT-BMT) Version 4 quality of life instrument into Arabic Language - English version

	PHYSICAL WELL-BEING	Not at all	A little bit	Some-what	Quite a bit	Very much
GP1	I have a lack of energy	0	1	2	3	4
GP2	I have nausea	0	1	2	3	4
GP3	Because of my physical condition, I have trouble meeting the needs of my family	0	1	2	3	4
GP4	I have pain	0	1	2	3	4
GP5	I am bothered by side effects of treatment	0	1	2	3	4
GP6	I feel ill	0	1	2	3	4
GP7	I am forced to spend time in bed	0	1	2	3	4
	SOCIAL/FAMILY WELL-BEING	Not at all	A little bit	Some-what	Quite a bit	Very much
GS1	I feel close to my friends	0	1	2	3	4
GS2	I get emotional support from my family	0	1	2	3	4
GS3	I get support from my friends	0	1	2	3	4
GS4	My family has accepted my illness	0	1	2	3	4
GS5	I am satisfied with family communication about my illness	0	1	2	3	4
GS6	I feel close to my partner (or the person who is my main support)	0	1	2	3	4
Q1	*Regardless of your current level of sexual activity, please answer the following question. If you prefer not to answer it, please mark this box and go to the next section.*					
GS7	I am satisfied with my sex life	0	1	2	3	4
	EMOTIONAL WELL-BEING	Not at all	A little bit	Some-what	Quite a bit	Very much
GE1	I feel sad	0	1	2	3	4
GE2	I am satisfied with how I am coping with my illness	0	1	2	3	4
GE3	I am losing hope in the fight against my illness	0	1	2	3	4
GE4	I feel nervous	0	1	2	3	4
GE5	I worry about dying	0	1	2	3	4
GE6	I worry that my condition will get worse	0	1	2	3	4
	FUNCTIONAL WELL-BEING	Not at all	A little bit	Some-what	Quite a bit	Very much
GF1	I am able to work (include work at home)	0	1	2	3	4
GF2	My work (include work at home) is fulfilling	0	1	2	3	4
GF3	I am able to enjoy life	0	1	2	3	4
GF4	I have accepted my illness	0	1	2	3	4
GF5	I am sleeping well	0	1	2	3	4
GF6	I am enjoying the things I usually do for fun	0	1	2	3	4
GF7	I am content with the quality of my life right now	0	1	2	3	4
	ADDITIONAL CONCERNS	Not at all	A little bit	Some-what	Quite a bit	Very much
BMT1	I am concerned about keeping my job (include work at home)	0	1	2	3	4
BMT2	I feel distant from other people	0	1	2	3	4
BMT3	I worry that the transplant will not work	0	1	2	3	4
BMT4	The effects of treatment are worse than I had imagined	0	1	2	3	4
C6	I have a good appetite	0	1	2	3	4
C7	I like the appearance of my body	0	1	2	3	4
BMT5	I am able to get around by myself	0	1	2	3	4
BMT6	I get tired easily	0	1	2	3	4
BL4	I am interested in sex	0	1	2	3	4
BMT7	I have concerns about my ability to have children	0	1	2	3	4
BMT8	I have confidence in my nurse(s)	0	1	2	3	4
BMT9	I regret having the bone marrow transplant	0	1	2	3	4
BMT10	I can remember things	0	1	2	3	4
Br1	I am able to concentrate	0	1	2	3	4
BMT11	I have frequent colds/infections	0	1	2	3	4
BMT12	My eyesight is blurry	0	1	2	3	4
BMT13	I am bothered by a change in the way food tastes	0	1	2	3	4
BMT14	I have tremors	0	1	2	3	4
B1	I have been short of breath	0	1	2	3	4
BMT15	I am bothered by skin problems (e.g., rash, itching)	0	1	2	3	4
BMT16	I have trouble with my bowels	0	1	2	3	4
BMT17	My illness is a personal hardship for my close family members	0	1	2	3	4
BMT18	The cost of my treatment is a burden on me or my family	0	1	2	3	4

Below is a list of statements that other people with your illness have said are important. Please circle or mark one number per line to indicate your response as it applies to the past 7 days

### Factorial validity (construct validity)

Using Factor analysis, the distribution of the questionnaire items among the predetermined factors(domains) were as follows: PWB (5/7), SWB (7/7), EWB (5/6), FWB (6/7) and BMT domain 6/23 items were belonging to their corresponding domains. However, in BMT domain, 11/23 items were distributed among 4 other dimensions. The test showed that 11/50 factors were belonging to no group. This is shown in Additional file 4. Factor loading using Equimax method. Results were almost the same using Varimax method.

### Internal consistency

Reliability coefficients Cronbach’s alpha indicated an excellent internal consistency (0.90); the domain values ranged from 0.67 to 0.91. [21] The lowest value was generated by item [16], which is GE2, in the EWB domain. After excluding this question, a small increase in the alpha value was seen (to 0.70), which suggested that this question had a weak correlation with the domain of emotional wellbeing (Table 4).

**Table 4:** Reliability measures for the Arabic-translated FACT-BMT questionnaire Subscales and total scales using Cronbach’s alpha and Intra-class correlation for consistency

FACT-BMT Subscales/ totals	Number	Cronbach’s alpha
Physical wellbeing (PWB)	104	0.84
Social wellbeing (SWB)	103	0.78
Emotional wellbeing (EWB)	104	0.67
Functional wellbeing (FWB)	103	0.88
Bone Marrow transplant (BMT)	104	0.81
FACT-General	102	0.89
FACT-BMT	104	0.90
FACT- Trial outcome Index (TOI)	103	0.91

### Quality of life assessment

Patients had scores in all domains of quality of life including physical, social, emotional and functional well-being, indicating that most patients were leading a normal life (Table 2). For PWB domain, 75% patients had not at all or a little bit issue related to lack of energy, nausea, trouble meeting family needs, having pain or feeling ill. In the SWB, excellent support from family and friends were noticed in 82% of the patients. EWB was excellent in 63% patients. In the FWB domain, 78% of the patients had very high scores. Similarly, in 23 additional BMT questions, we have observed excellent scores. This is more clearly demonstrated in Table 5 (clinical scores). Scoring and clinical interpretation require caution as all the items in SWB and FWB, one item in EWB (GE2) and selected items in BMT domain (C6,C7, BMT5, BL4,BMT8) are reverse items.

**Table 5:** Clinical scores of FACT-BMT domains (In percentage-total of 108 patients)

FACT-BMT Subscales	Not at all	A little bit	Some-what	Quite a bit	Very much
Physical wellbeing (PWB)	59.5	15.5	13	8	4
Social wellbeing (SWB)	3.6	4.3	10.3	25.6	56.1
Emotional wellbeing (EWB)	53.9	9.5	13.2	12.5	10.9
Functional wellbeing (FWB)	2.7	3.1	16	30.8	47.5
FACT-BMT	42.5	11.8	13.5	14.8	17.4

#### Seventeen extra questions

Seventeen extra questions focused on three main areas: decision making for the treatment, use of alternative treatments and family and social support.

Decision making: During HDC ASCT decision making, 51% patients made the decision by themselves. Patients + other family member/s made the decision in 31%, and in 16 (15%) cases, this was by the family member/s only; 8 male patients (7 single and 1 married, all < 26 age (6 were < 20) and 8 female patients (5 married, 2 single, 1 widowed, ages 17–37 in 6). Treating physician was among the decision makers with the patient + the family members in 6% cases.

Alternative treatment: Alternative treatment was used by 72%; of these 72%, most commonly used treatment was honey in 91%, black seeds (*Nigella sativa* / kezah / habbat albarakah / kalonji) in 73%, Zam Zam (holy water from Makkah) 77%, camel urine alone in 15%, camel milk alone in 3% or combining camel milk + camel urine in 8%, making a total of camel product use of 26%. Sixty-six percent patients sought counseling with some kind of spiritual counselor (Sheikh to read Quran (the Holy Muslim Book)) for healing or make special supplication for them.

Family and social support: More than 90% reported good support from family and friends; relationship with parents (99%), spouse (93%) and children (91%) was not affected due to HDC ASCT. No patient (74 eligible patients) faced separation from spouse due this condition. Getting married was considered difficult in 27% of eligible patients. Young patients observed significant issues related to their education. Twenty-seven patients (37%) mentioned that their education was affected and 58% reported that their educational institution was not supportive during this time. Six (10%) patients were unable to get admission in any educational institution as a result of their illness / absences / failure to get scholarships despite their efforts. Employer either fired or stopped 11% of patients from working and another 22% stopped working electively due to their condition. Finding a new job was difficult due to this condition in 23%.

## Discussion

There are no validated and reliable tools to assess QoL for Arabic speaking patients undergoing HDC ASCT. This becomes increasingly more important since HDC ASCT is being increasingly offered to patients with relapsed and refractory aggressive NHL and HL. Psychological and physical problems affecting QoL due to BMT have been reported in the past [22]. Since a significant proportion of these patients are long term survivors, QoL has gained significant importance as an assessment measure following HDC ASCT. FACT-BMT is a multifunctional tool allowing a comprehensive assessment of overall QoL. The initial version was developed in English. In view of cultural variation among different societies, it is important to adapt QoL assessment tool according to the local culture and validate it for the language of the patients answering the questionnaire. For this reason, studies assessing QoL have been slow among non-English speakers. This holds particularly true for Arabic speaker since Arabic Translation and validation of QoL tools has been rare with few exceptions [23–25]. This study thus provides an unmet need for the Arabic speaking population undergoing HDC and ASCT by translating and validating the FACT-BMT version [4] into the Arabic language. Our translation process strictly followed the FACIT guidelines. Cronbach’s alpha of greater than 0.70 and TOI of 0.90 signify validity and reliability of Arabic translation of FACT-BMT. We have, therefore, attempted and provided a reliable and validated tool for assessment of the quality of life among patients undergoing BMT for Arabic speaking patients.

Following statistical validation of Arabic version of FACT-BMT version 4, we continued QoL assessment among 108 who underwent HDC ASCT at our center. These patients had either DLBCL or HL and received uniform treatment in our institution. None of these patients underwent allogeneic transplant which has a very different spectrum of both short and long term effects on patient’s QoL due to significantly higher morbidity related to graft versus host disease, hepatic veno-occlusive disease as well as side effects to port transplant therapies. There are significantly more reports on QoL in patients undergoing allogeneic transplant [26–33].

In our study, patients from all the regions in the kingdom of Saudi Arabia were included, providing a good representation within the same country. Only 7 patients (6.9%) had no formal education and required assistance while answering the questionnaire. Major limitations of our study is lack of QoL assessment prior to HDC ASCT that could have served as a baseline to compare the impact of HDC at various time frames like one, six and twenty-four months. Another concern is the absence of QoL reporting at pre-specified intervals post-HDC ASCT. However, 54% of the patients had their QoL assessment within two years from the transplant. Another limitation is relatively small number of patients (108), inclusion of patients with DLBCL or HL only that limits the understanding of QoL in other hematological conditions requiring HDC ASCT and lack of planned interim analysis. We found no differences among scores for different FACT-BMT domains when tested for time since transplant. Future studies may focus on QoL at various pre-specified time frames especially long term (after five years) effects in the group which was not sexually active at the time of HDC ASCT.

Our results revealed that our patients had high scores in all domains of quality of life including physical, social, emotional and functional well-being, indicating that most patients were leading a normal life. We tested seventeen additional issues felt to be important in Arab culture and believed to provide insight into decision making, relationship with family members, educational institution, employment related issues and use of alternative treatments. During HDC ASCT about half of the patients made HDC ASCT decision alone. Large number of patients used alternative treatment without informing their physicians. Most patients reported excellent support from the family and the friends. Educational and related support system was suboptimal. Also, employment related issues need to be addressed thoroughly.

## Conclusion

Our data validate the first Arabic version of FACT-BMT (Version 4) and will help large numbers of Arabic speaking patients, not only in the Middle East and Arab countries, but also in non-Arabic speaking areas of the world. Our results indicate that most of our patients enjoy excellent social and family support and a good QoL post-HDC ASCT. In the Middle East and Arab countries, this is the first study which objectively assessed family and social support issues. These patients had good family support from close relatives. There is a significant need to optimize appropriate social support in our community for these patients; especially from educational institutions and employers. Enough social support to highly vulnerable transplant patients may improve QoL post-HDC ASCT.

## Additional files

Additional file 1: Translation and validation of the Functional Assessment of Cancer Therapy-Bone Marrow Transplant (FACT-BMT) Version 4 quality of life instrument into Arabic Language. (DOC 139 kb)

Additional file 2: Cultural questions – Arabic (neither part nor related to FACT-BMT) (DOC 85 kb)

Additional file 3: Cultural questions – English (neither part nor related to FACT-BMT) (DOC 75 kb)

Additional file 4: Factor loading using Equimax method (DOCX 20 kb)
