# Supplementary material for: Translation and validation of the Functional Assessment of Cancer Therapy-Bone Marrow Transplant (FACT-BMT) version 4 quality of life instrument into Arabic language
Source: Health Qual Life Outcomes. 2018 Mar 12;16:47. doi: 10.1186/s12955-018-0861-7 (PMC5848601; doi:10.1186/s12955-018-0861-7)
Supplement: Supplementary file 1 — Translation and validation of the Functional Assessment of Cancer Therapy-Bone Marrow Transplant (FACT-BMT) Version 4 quality of life instrument into Arabic Language. (DOC 139 kb) [file 12955_2018_861_MOESM1_ESM.doc]

**Additional file 1. Translation and validation of the Functional Assessment of Cancer Therapy-Bone Marrow Transplant (FACT-BMT) Version 4 quality of life instrument into Arabic Language**

هذه قائمة بالعبارات التي ذكر أشخاص بنفس علتك أنها مهمة بالنسبة لهم. من فضلك ضع دائرة أو علامة على رقم واحد في كل سطر لبيان مدى انطباق إجابتك على حالتك في الأيام السبعة الأخيرة.

|  | **الكفاءة الجسمانية** | **ليس على الإطلاق** | **مرات قليلة** | **نوعاً ما** | **غالبا** | **كثيرا جدا** |
| --- | --- | --- | --- | --- | --- | --- |
|  |
|  |
| GP1 | أشعر بالوهن | 0 | 1 | 2 | 3 | 4 |
| GP2 | أشعر بالغثيان | 0 | 1 | 2 | 3 | 4 |
| GP3 | بسبب حالتي الصحية – لدي صعوبة في تلبية احتياجات أسرتي | 0 | 1 | 2 | 3 | 4 |
| GP4 | أشعر بألم | 0 | 1 | 2 | 3 | 4 |
| GP5 | أشعر بالضيق من الآثار الجانبية للعلاج | 0 | 1 | 2 | 3 | 4 |
| GP6 | أشعر أنني عليل (مريض) | 0 | 1 | 2 | 3 | 4 |
| GP7 | أنا مضطر لملازمة الفراش | 0 | 1 | 2 | 3 | 4 |
|  |  |  |  |  |  |  |
|  | **الكفاءة الاجتماعية و الأسرية** | **ليس على الإطلاق** | **مرات قليلة** | **نوعاً ما** | **غالبا** | **كثيرا جدا** |
|  |
|  |
| GS1 | أشعر أنني قريب من أصدقائي | 0 | 1 | 2 | 3 | 4 |
| GS2 | أحظى بتعاطف أسرتي | 0 | 1 | 2 | 3 | 4 |
| GS3 | أجد كل الدعم من أصدقائي | 0 | 1 | 2 | 3 | 4 |
| GS4 | لقد تقبلت أسرتي حالتي الصحية | 0 | 1 | 2 | 3 | 4 |
| GS5 | أنا راض عن الكيفية التي تتعامل بها أسرتي مع مرضي | 0 | 1 | 2 | 3 | 4 |
| GS6 | أشعر بالقرب من زوجي / زوجتي | 0 | 1 | 2 | 3 | 4 |
| Q1 | **بغض النظر عن حالتك الجنسية حاليا، من فضلك أجب عن هذا السؤال. إذا كنت لا ترغب في الإجابة من فضلك ضع علامة في هذا المربع 🞎 ثم انتقل إلى السؤال التالي:** | | | | | |
| GS7 | أنا راض عن حياتي الجنسية | 0 | 1 | 2 | 3 | 4 |

من فضلك ضع دائرة أو علامة على رقم واحد في كل سطر لبيان مدى انطباق إجابتك على حالتك في الأيام السبعة الأخيرة**.**

|  | **الكفاءة العاطفـــــية** | **ليس على الإطلاق** | **مرات قليلة** | **نوعاً ما** | **غالبا** | **كثيرا جدا** |
| --- | --- | --- | --- | --- | --- | --- |
|  |
|  |
| GE1 | أشعر بالحزن | 0 | 1 | 2 | 3 | 4 |
| GE2 | أنا راض عن تقبلي لحالتي الصحية | 0 | 1 | 2 | 3 | 4 |
| GE3 | أفتقد الأمل في مقاومتي لمرضي | 0 | 1 | 2 | 3 | 4 |
| GE4 | أشعر بالعصبية | 0 | 1 | 2 | 3 | 4 |
| GE5 | أنا قلق من الموت | 0 | 1 | 2 | 3 | 4 |
| GE6 | أخاف أن تسوء حالتي | 0 | 1 | 2 | 3 | 4 |

|  | **الكفاءة الوظيفيـــــــــة** | **ليس على الإطلاق** | **مرات قليلة** | **نوعاً ما** | **غالبا** | **كثيرا جدا** |
| --- | --- | --- | --- | --- | --- | --- |
|  |
|  |
| GF1 | أنا قادر على العمل (بما في ذلك العمل في المنزل) | 0 | 1 | 2 | 3 | 4 |
| GF2 | عملي (بما في ذلك عملي في المنزل) يرضيني | 0 | 1 | 2 | 3 | 4 |
| GF3 | أنا قادر على الاستمتاع بالحياة | 0 | 1 | 2 | 3 | 4 |
| GF4 | لقد تقبلت مرضي | 0 | 1 | 2 | 3 | 4 |
| GF5 | أنام جيدا | 0 | 1 | 2 | 3 | 4 |
| GF6 | أستمتع بالأشياء التي أقوم بها للترفيه | 0 | 1 | 2 | 3 | 4 |
| GF7 | أنا راض عن طبيعة حياتي الآن | 0 | 1 | 2 | 3 | 4 |

من فضلك ضع دائرة أو علامة على رقم واحد في كل سطر لبيان مدى انطباق إجابتك على حالتك في الأيام السبعة الأخيرة**.**

|  | اهتمامات إضافية | **ليس على الإطلاق** | **مرات قليلة** | **نوعاً ما** | **غالبا** | **كثيرا جدا** |
| --- | --- | --- | --- | --- | --- | --- |
|  |
|  |
| BMT1 | أنا قلق بشأن الحفاظ على وظيفتي  ( متضمناً العمل بالبيت) | 0 | 1 | 2 | 3 | 4 |
| BMT2 | أنا أشعر بأني بعيداً عن الأخرين  ( متضمناًً العمل بالبيت) | 0 | 1 | 2 | 3 | 4 |
| BMT3 | أنا قلق بأن الزراعة لن تعمل | 0 | 1 | 2 | 3 | 4 |
| BMT4 | تأثيرات العلاج الجانبية كانت أسوأ مما تخيليت | 0 | 1 | 2 | 3 | 4 |
| C6 | لدي شهية جيدة | 0 | 1 | 2 | 3 | 4 |
| C7 | أنا راض عن شكل جسمي | 0 | 1 | 2 | 3 | 4 |
| BMT5 | عندي القدرة على التحرك والتنقل بمفردي | 0 | 1 | 2 | 3 | 4 |
| BMT6 | أتعب بسهولة | 0 | 1 | 2 | 3 | 4 |
| BL4 | أحب ممارسة الجنس 0 | 0 | 1 | 2 | 3 | 4 |
| BMT7 | عندي قلق بخصوص قدرتي على إنجاب أطفال | 0 | 1 | 2 | 3 | 4 |
| BMT8 | أنا لدي أطمئنان من ممرضاتي | 0 | 1 | 2 | 3 | 4 |
| BMT9 | أنا ندمت لأني زرعت نخاع العظم | 0 | 1 | 2 | 3 | 4 |
| BMT10 | أنا أستطيع تذكر الأشياء | 0 | 1 | 2 | 3 | 4 |
| Br1 | أنا قادر على التركيز ( مثال، القراءة) | 0 | 1 | 2 | 3 | 4 |
| BMT11 | أنا أعاني من التهابات متكررة/ نزلة برد متكررة | 0 | 1 | 2 | 3 | 4 |
| BMT12 | أعاني من غشاوة فى البصر | 0 | 1 | 2 | 3 | 4 |
| BMT13 | أنا منزعج من تغير طعم الأكل 0 | 0 | 1 | 2 | 3 | 4 |
| BMT14 | أنا عندي رجفة باليد 0 | 0 | 1 | 2 | 3 | 4 |
| B1 | أعاني من نوبات ضيق تنفس 0 | 0 | 1 | 2 | 3 | 4 |
| BMT15 | أنا منزعج من مشكلة جلدية ( طفح ، حكة) 0 | 0 | 1 | 2 | 3 | 4 |
| BMT16 | أنا عندي اضطراب في الأمعاء 0 | 0 | 1 | 2 | 3 | 4 |
| BMT17 | مرضي حمل أعضاء عائلتي مشقة 0 | 0 | 1 | 2 | 3 | 4 |
| BMT18 | تكلفة علاجي تشكل عبئاً على عائلتي 0 | 0 | 1 | 2 | 3 | 4 |
