# Supplementary material for: Translation and validation of the Functional Assessment of Cancer Therapy-Bone Marrow Transplant (FACT-BMT) version 4 quality of life instrument into Arabic language
Source: Health Qual Life Outcomes. 2018 Mar 12;16:47. doi: 10.1186/s12955-018-0861-7 (PMC5848601; doi:10.1186/s12955-018-0861-7)
Supplement: Supplementary file 2 — Cultural questions – Arabic (neither part nor related to FACT-BMT) (DOC 85 kb) [file 12955_2018_861_MOESM2_ESM.doc]

**Additional file 2. Cultural questions – Arabic (neither part nor related to FACT-BMT)**

أسئلة خاصة بالمجتمع

|  | الاسئلة | **لا** | **نعم** | **غير ملائم للتطبيق** |
| --- | --- | --- | --- | --- |
| 1 | معظم أقربائي عندهم فكرة كاملة عن حالتي ⁄ علاجي |  |  |  |
| 2 | أنا عندي مشكلة للحصول على زوج(زوجة ) بسبب مرضي (للمرضى:الأعزب،المطلق،الأرمل). |  |  |  |
| 3 | للمرضى الذي كانوا يعملون قبل الزراعة أنا فصلت من عملي ⁄ أجبرت على ترك عملي بسبب مرضي |  |  |  |
| 4 | أنا تركت عملي باختياري |  |  |  |
| 5 | أنا لدي مشكلة في الحصول على وظيفة جديدة بسبب مرضي وعلاجي |  |  |  |
| 6 | تحصيلي العلمي تأثر بسبب هذه المشكلة |  |  |  |
| 7 | قسم الخدمة الاجتماعية في مستشفى الملك فيصل التخصصي ومركز الأبحاث قدم لي المساعدة والدعم |  |  |  |
| 8 | الهيئة التعليمية (مدرستي،جامعتي،معهدي) قدمت لي الدعم خلال فترة مرضي وأثناء فترة الزراعة |  |  |  |
| 9 | أنا لم أستطع الحصول على القبول في هيئة تعليمية الآن بسبب مرضي |  |  |  |
| 10 | مرضي تسبب انفصالي عن شريك حياتي |  |  |  |
| 11 | علاقتي بقيت مستقرة مع |  |  |  |
|  | الأب |  |  |  |
|  | الأم |  |  |  |
|  | الزوج |  |  |  |
|  | أطفالي |  |  |  |
| 12 | من هو الذي اتخذ قرار الزراعة |  |  |  |
|  | المريض |  |  |  |
|  | الزوج 0 |  |  |  |
|  | الأخ 0 |  |  |  |
|  | الأب 0 |  |  |  |
|  | الأم 0 |  |  |  |
|  | العم 0 |  |  |  |
|  | آخرون 0 |  |  |  |
| 13 | هل استخدمت العلاج البديل…………………………. |  |  |  |
|  | إذا كانت الإجابة بنعم |  |  |  |
|  | عسل……………………………………………………… |  |  |  |
|  | الحبة السوداء…………………………………………… |  |  |  |
|  | ماء زمزم………………………………………………… |  |  |  |
|  | بول الجمل………………………………………… |  |  |  |
|  | أخرى………………………………………………… |  |  |  |
| 14 | أنا قمت بمراجعة شيخ للعلاج……………………… |  |  |  |
| 15 | لقد انجبت بعد ان زرعت النخاع العظم (للرجال)………… |  |  |  |
| 16 | لقد انجبت بعد ان زرعت نخاع العظم (للنساء)…………… |  |  |  |
| 17 | انا كنت سوف ارفض الزراعة لوعلمت بكل مضاعفاتها…… |  |  |  |
