# Supplementary material for: Translation and validation of the Functional Assessment of Cancer Therapy-Bone Marrow Transplant (FACT-BMT) version 4 quality of life instrument into Arabic language
Source: Health Qual Life Outcomes. 2018 Mar 12;16:47. doi: 10.1186/s12955-018-0861-7 (PMC5848601; doi:10.1186/s12955-018-0861-7)
Supplement: Supplementary file 3 — Cultural questions – English (neither part nor related to FACT-BMT) (DOC 75 kb) [file 12955_2018_861_MOESM3_ESM.doc]

**Additional file 3. Cultural questions – English (neither part nor related to FACT-BMT)**

**CULTURAL ADAPTED QUESTIONS**

**Please circle mark one choice per question to indicate your response**

|  | QUESTIONS | **No** | | **Yes** | **NA** |
| --- | --- | --- | --- | --- | --- |
| 1 | Most of my close family members are aware of my condition\treatment | |  |  |  |
| 2 | I am having problem (had problem) getting married due to this condition (those who were single\divorced\widowed) | |  |  |  |
| 3 | Those who were working before HDC: I was fired\forced to quit my job due to this condition | |  |  |  |
| 4 | I electively quit my job | |  |  |  |
| 5 | I am having problem finding a new job due to my condition and treatment | |  |  |  |
| 6 | My education was affected due to this problem | |  |  |  |
| 7 | Social service of KFSH RC provided help \ support in my care | |  |  |  |
| 8 | My education institution supported me during this time | |  |  |  |
| 9 | I am unable to get admission in education institution now due my condition | |  |  |  |
| 10 | My condition caused my partner to separate | |  |  |  |
| 11 | My relationship remained unchanged with | |  |  |  |
|  | Father | |  |  |  |
|  | Mother | |  |  |  |
|  | Spouse | |  |  |  |
|  | Kids | |  |  |  |
| 12 | Who made the decision of BMT | |  |  |  |
|  | Patient | |  |  |  |
|  | Spouse | |  |  |  |
|  | Brother | |  |  |  |
|  | Father | |  |  |  |
|  | Mother | |  |  |  |

|  |  | **No** | | **Yes** | **NA** |
| --- | --- | --- | --- | --- | --- |
|  | Uncle | |  |  |  |
|  | Others……………………………………………………………………… | |  |  |  |
| 13 | Alternative treatment | |  |  |  |
|  | IF YES, THEN MARK FROM THE OPTIONS BELOW | |  |  |  |
|  | Honey | |  |  |  |
|  | Black seed | |  |  |  |
|  | Zam Zam | |  |  |  |
|  | Camel urine………………………………………………………………... | |  |  |  |
|  | Others | |  |  |  |
| 14 | Consulted spiritual advisor (sheikh) for treatment | |  |  |  |
| 15 | Did you have children after high dose chemotherapy and stem cell transplant (For Males) | |  |  |  |
| 16 | Did you have children after high dose chemotherapy and stem cell transplant (For Females) | |  |  |  |
| 17 | Would you have rejected BMT if you knew the associated complications ………………………….…………………………………… | |  |  |  |
