# Supplementary material for: Translation and validation of the Functional Assessment of Cancer Therapy-Bone Marrow Transplant (FACT-BMT) version 4 quality of life instrument into Arabic language
Source: Health Qual Life Outcomes. 2018 Mar 12;16:47. doi: 10.1186/s12955-018-0861-7 (PMC5848601; doi:10.1186/s12955-018-0861-7)
Supplement: Supplementary file 4 — Factor loading using Equimax method (DOCX 20 kb) [file 12955_2018_861_MOESM4_ESM.docx]

|  | **Additional file 4. Factor loading using Equimax method** | | | | | |
| --- | --- | --- | --- | --- | --- | --- |
| **Rotated Component Matrix^a^** | | | | | |  |
|  | GP | GF | GS | BMT | GE |  |
|  | 1 | 2 | 3 | 4 | 5 |  |
| GP1 | **0.745** | -0.138 | -0.103 | 0.154 | 0.11 |  |
| GP2 | **0.753** | -0.067 | 0.145 | 0.154 | -0.148 |  |
| GP3 | 0.115 | -0.143 | 0.024 | **0.68** | 0.032 | 5/7 |
| GP4 | **0.764** | -0.129 | -0.038 | 0.169 | 0.322 |  |
| GP5 | **0.721** | -0.077 | -0.066 | 0.194 | 0.185 |  |
| GP6 | **0.635** | -0.278 | 0.125 | 0.273 | 0.373 |  |
| GP7 | 0.451 | **-0.466** | 0.29 | 0.391 | 0.076 |  |
| GS1 | -0.141 | 0.129 | **0.738** | -0.403 | -0.29 |  |
| GS2 | 0.053 | 0.125 | **0.78** | -0.063 | 0.061 |  |
| GS3 | 0.142 | 0.344 | **0.658** | -0.092 | -0.039 |  |
| GS4 | -0.246 | 0.324 | **0.481** | 0.111 | -0.039 | 7/7 |
| GS5 | -0.081 | -0.084 | **0.619** | -0.154 | -0.104 |  |
| GS6 | 0.201 | 0.48 | **0.572** | -0.024 | 0.153 |  |
| GS7 | -0.111 | 0.065 | **0.389** | 0.014 | 0.159 |  |
| GE1 | 0.383 | -0.068 | -0.107 | 0.246 | **0.597** |  |
| GE2 | -0.133 | 0.261 | -0.033 | 0.237 | **-0.283** |  |
| GE3 | -0.053 | -0.062 | -0.037 | -0.111 | **0.614** | 5/6 |
| GE4 | **0.448** | -0.013 | -0.079 | 0.244 | 0.193 |  |
| GE5 | -0.226 | 0.058 | 0.2 | 0.188 | **0.541** |  |
| GE6 | 0.019 | -0.406 | -0.091 | -0.105 | **0.616** |  |
| GF1 | -0.313 | **0.65** | 0.015 | -0.334 | 0.01 |  |
| GF2 | -0.191 | **0.776** | 0.098 | -0.349 | -0.101 |  |
| GF3 | -0.075 | **0.749** | 0.215 | -0.03 | -0.218 |  |
| GF4 | -0.074 | **0.55** | 0.338 | 0.265 | -0.385 | 6/7 |
| GF5 | **-0.665** | **0.361** | 0.334 | 0.074 | -0.124 |  |
| GF6 | 0.055 | **0.69** | 0.473 | -0.064 | -0.04 |  |
| GF7 | -0.092 | **0.588** | 0.454 | 0.037 | -0.327 |  |
| BMT1 | -0.067 | -0.02 | -0.071 | 0.142 | **0.782** |  |
| BMT2 | 0.075 | -0.018 | -0.012 | **0.477** | 0.38 |  |
| BMT3 | -0.133 | -0.385 | 0.09 | **0.571** | -0.057 |  |
| BMT4 | **0.59** | -0.513 | -0.146 | 0.127 | 0.159 |  |
| C6 | **-0.728** | 0.357 | 0.213 | -0.134 | -0.064 |  |
| C7 | -0.172 | **0.652** | 0.194 | -0.062 | 0.012 |  |
| BMT5 | -0.215 | **0.665** | -0.12 | -0.291 | -0.181 |  |
| BMT6 | **0.638** | -0.183 | 0.003 | 0.189 | 0.403 |  |
| BL4 | 0.09 | 0.369 | **0.448** | 0.165 | 0.268 | 6/22 |
| BMT7 | 0.291 | -0.066 | -0.053 | **-0.389** | 0.383 |  |
| BMT8 | **-0.606** | 0.418 | 0.041 | 0.05 | 0.032 |  |
| BMT9 | 0.009 | -0.337 | 0.316 | **0.471** | 0.423 |  |
| BMT10 | -0.175 | 0.164 | 0.082 | **-0.641** | -0.079 |  |
| Br1 | -0.277 | -0.001 | 0.285 | **-0.642** | -0.122 |  |
| BMT11 | **0.471** | 0.01 | -0.21 | 0.413 | 0.145 |  |
| BMT13 | **0.449** | 0.121 | -0.16 | 0.347 | -0.06 |  |
| BMT14 | **0.811** | -0.236 | 0.082 | 0.107 | -0.141 |  |
| B1 | **0.515** | -0.022 | -0.37 | 0.323 | 0.498 |  |
| BMT15 | 0.269 | 0.048 | -0.283 | **0.545** | -0.023 |  |
| BMT16 | **0.77** | -0.135 | -0.064 | 0.274 | -0.078 |  |
| BMT17 | 0.127 | -0.018 | -0.048 | 0.393 | **0.437** |  |
| BMT18 | -0.005 | 0.085 | **-0.651** | 0.045 | 0.302 |  |
| Extraction Method: Principal Component Analysis.  Rotation Method: **Equamax with Kaiser Normalization.** | | | | | |  |
| a Rotation converged in 19 iterations. | | | | | |  |
